# Critical role of mitogen-inducible gene 6 in restraining endothelial cell permeability to maintain vascular homeostasis

**DOI:** 10.1007/s12079-022-00704-z

**Published:** 2022-10-25

**Authors:** Liying Xing, Guanqun Huang, Rongyuan Chen, Lijuan Huang, Juanxi Liu, Xiangrong Ren, Shasha Wang, Haiqing Kuang, Anil Kumar, Jong Kyong Kim, Qin Jiang, Xuri Li, Chunsik Lee

**Affiliations:** 1 State Key Laboratory of Ophthalmology, Zhongshan Ophthalmic Center, Guangdong Provincial Key Laboratory of Ophthalmology and Visual Science, Sun Yat-sen University, Guangzhou, 510060 China; 2 Affiliated Eye Hospital of Nanjing Medical University, Nanjing, 210000 China

**Keywords:** MIG6, Vascular permeability, VEGFR2, Endothelial cell barrier

## Abstract

**Abstract:**

Although mitogen-inducible gene 6 (MIG6) is highly expressed in vascular endothelial cells, it remains unknown whether MIG6 affects vascular permeability. Here, we show for the first time a critical role of MIG6 in limiting vascular permeability. We unveil that genetic deletion of *Mig6* in mice markedly increased VEGFA-induced vascular permeability, and MIG6 knockdown impaired endothelial barrier function. Mechanistically, we reveal that MIG6 inhibits VEGFR2 phosphorylation by binding to the VEGFR2 kinase domain 2, and MIG6 knockdown increases the downstream signaling of VEGFR2 by enhancing phosphorylation of PLCγ1 and eNOS. Moreover, MIG6 knockdown disrupted the balance between RAC1 and RHOA GTPase activation, leading to endothelial cell barrier breakdown and the elevation of vascular permeability. Our findings demonstrate an essential role of MIG6 in maintaining endothelial cell barrier integrity and point to potential therapeutic implications of MIG6 in the treatment of diseases involving vascular permeability.

**Graphical abstract:**

Xing *et al*. (2022) investigated the critical role of MIG6 in vascular permeability. MIG6 deficiency promotes VEGFA-induced vascular permeability via activation of PLCγ1-Ca^2+^-eNOS signaling and perturbation of the balance in RAC1/RHOA activation, resulting in endothelial barrier disruption.
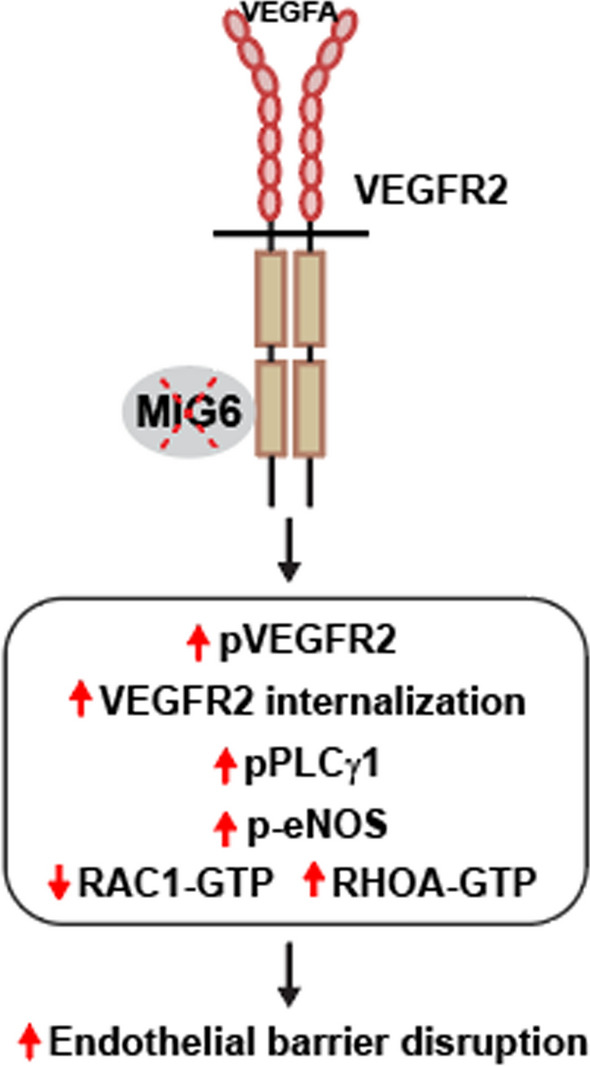

**Supplementary Information:**

The online version contains supplementary material available at 10.1007/s12079-022-00704-z.

## Introduction

Endothelial cells (EC) line the luminal side of blood vessels and form a semi-permeable barrier between blood and interstitial space (Aird 2007; Boulanger 2016; Dalal et al. 2020). This selective barrier is a highly complex and orchestrated structure that controls movement of electrolytes, proteins, and cells across intact endothelial barrier via transcellular and paracellular pathways. The disruption of endothelial barrier causes aberrant elevation of vascular permeability, which is highly prevalent in pathological conditions such as cancer, stroke, and diabetic retinopathy (Bates 2010; Park-Windhol and D'Amore 2016). EC barrier function is modulated by different types of EC junctions such as gap junctions (GJ), tight junctions (TJ), and adherens junctions (AJ) (Komarova et al. 2017; Radeva and Waschke 2018). In particular, stability of endothelial cell–cell contacts and permeability for large molecules are provided by AJ. VE-cadherin is considered to be the main component of AJ integrity (Duong and Vestweber 2020), and Ca^2+^-dependent-homophilic binding of VE-cadherin to endothelial cell–cell junction forms zipper-like AJ along the cell contacts. Endothelial nitric oxide synthase (eNOS) also plays a critical role in the regulation of these intercellular junctions by contributing to the maintenance of endothelial barrier integrity (Komarova et al. 2017). Particularly, cytoskeletal reorganization accompanying endothelial permeability alterations is linked to eNOS activation (Di Lorenzo et al. 2013; Flentje et al. 2019; Su et al. 2005). Moreover, multiple VEGFA-mediated pathways involving SRC, AMP-activated protein kinase (AMPK), focal adhesion kinase (FAK), and phospholipase C gamma (PLCγ) activation are known to affect the EC barrier and vascular permeability (Dragoni et al. 2021; Komarova et al. 2017).

VEGFR2 undergoes phosphorylation at various tyrosine (Tyr) and serine/threonine (Ser/Thr) residues. There are major Tyr phosphorylation sites on VEGFR2, which participate in VEGF responses to regulate EC proliferation, migration, permeability, and survival (Shaik et al. 2020; Simons et al. 2016; Wang et al. 2020). Of note, phosphorylated Tyr951 (pY951) serves as a binding site for T-cell specific adaptor protein (TSad) to control EC migration and permeability, and both pY1054 and pY1059 located in the activation loop of kinase domain 2 play critical roles for kinase activity (Koch and Claesson-Welsh 2012; Sun et al. 2012). Additionally, pY1175 and pY1214 also create the binding sites for PLCγ1, SHB, SHC, GRB2, FYN, and NCK to activate downstream signaling molecules (Koch and Claesson-Welsh 2012). Therefore, VEGFR2 phosphorylation is the key process to recruit the signaling molecules to VEGFR2, which can induce various biological responses.

Mitogen-inducible gene 6 (MIG6) is a ubiquitously expressed adaptor protein which was identified as a glucocorticoid-induced transcript from the rat liver (Lee et al. 1985; Xu and Li 2021). MIG6 is induced by a wide variety of extracellular stimuli including various growth factors, hormones, and cellular stress. It contains several domains for protein–protein interaction which is functionally critical for converting diverse signals to appropriate cellular responses. Importantly, MIG6 has been known as a negative feedback regulator of EGFR to restrain its oncogenic activity (Ferby et al. 2006; Hackel Peter et al. 2001). Moreover, it is also involved in many pathological conditions such as diabetes, cardiovascular diseases, psoriasis, and tumor progression (Xu and Li 2021). We have recently reported that MIG6 has anti-angiogenic effects in retinal angiogenesis and hypoxia-induced retinal neovascularization (Liu et al. 2021). In the aforementioned study, we demonstrated that *Mig6* knockout mice promote retinal angiogenesis and overexpression of MIG6 in ECs decreases microvessel outgrowth. This anti-angiogenic effect of MIG6 is mediated by binding to SHC1 and inhibiting its phosphorylation. Although we revealed that MIG6 has the anti-angiogenic effect, it remains unknown whether MIG6 can inhibit VEGFA-induced VEGFR2 signaling, which would subsequently affect vascular permeability along with other VEGFR2-mediated biological responses.

In this study, using *Mig6* knockout mice and cultured endothelial cells, we examined the effect of MIG6 modulation on vascular permeability. We show that MIG6 is a critical regulator in maintaining the integrity of endothelial barrier. *Mig6* knockout mice enhanced VEGFA-induced vascular leakage, and EC permeability was compromised by MIG6 knockdown. Importantly, MIG6 binds to the VEGFR2 kinase domain, and tyrosine phosphorylation on VEGFR2 is increased in MIG6 knockdown ECs. Notably, phosphorylation of PLCγ1 and eNOS was increased with a transient rise in Ca^2+^ in MIG6 knockdown ECs, suggesting that MIG6-mediated modulation of VEGFR2 phosphorylation negatively regulates the downstream effectors of the VEGFR2 signaling pathway. Moreover, MIG6 knockdown interrupted the fine balance between RHOA and RAC1 activation, leading to endothelial barrier breakdown and increased vascular permeability. These results altogether suggest that MIG6 is a critical molecule in the maintenance of EC barrier homeostasis.

## Materials and methods

### Mice

The *Mig6*-deficient mice were described previously (Zhang et al. 2005). All animal experiments were reviewed and approved by the Animal Use and Care Committee of Zhongshan Ophthalmic Center, Sun Yat-sen University. All animal procedures were done in compliance with the approved guideline.

### Cell culture and reagents

Primary human umbilical vein endothelial cells (HUVECs) were purchased from Angio-Proteomie (Boston, MA, USA). HUVECs were cultured in endothelial cell medium (ScienCell Research, Carlsbad, CA, USA) containing endothelial cell growth supplement (ECGS), 5% FBS, and penicillin/streptomycin (ScienCell Research). VEGFR inhibitor Axitinib (S1005, Selleck Chemicals, Shanghai, China), PLCγ1 inhibitor U73122 (S8011, Selleck Chemicals, Shanghai, China), and L-NG-nitroarginine methyl ester (L-NAME; S2877, Selleck Chemicals, Shanghai, China) were reconstituted in DMSO before use. Recombinant human VEGFA165 protein and histamine were purchased from PeproTech (100-20, Rocky Hill, NJ, USA) and MedChem Express (HY-B1204, Shanghai, China), respectively.

### Miles assay

Mice were subjected to intraperitoneal injection with pyrilamine maleate salt (4 mg/kg body weight in 0.9% saline, Selleckchem, Houston, TX, USA) to inhibit histamine release. Mice were injected with 200 μl Evans blue (0.5% Evans blue in sterile saline, Sigma) in the lateral tail vein and incubated for 1 h. VEGFA165 (50 ng in 10 μl/site) or BSA in sterile saline was injected intradermally and incubated for 1 h. Mice were euthanized by cervical dislocation, and then, the ear was dissected and dried at 56°C. After incubation with deionized formamide at 56ºC for overnight, the amount of Evans blue in each tissue sample was quantified by spectrometry at 620 nm (Molecular Devices).

### siRNA knockdown and viral infection of endothelial cells

For gene knockdown, ECs were transfected with siRNAs for the indicated genes or non-targeting scrambled negative control (Ribobio, Guangzhou, China) using ESCORT transfection reagent (L3287, Sigma). The siRNA sequences are listed in Supplementary Table 1. For adenoviral infection, ECs were infected with Ad-MIG6 (VH894726, Vigene Biosciences, Rockville, MD, USA) or Ad-GFP (CV10001, Vigene Biosciences) at an MOI of 10 for 48 h. Similarly, lentiviral transduction of ECs was performed with lenti-RAC1 (Q61L) (CH854034) or lenti-GFP (CV10002) purchased from Vigene Biosciences.

### Antibodies for western blots

Antibodies used in western blots are as follows: β-actin (RM2001, Ray Antibody Biotech, Beijing, China), α-tubulin (RM2007, Ray Antibody Biotech, Beijing, China), MIG6 (WH0054206M1, Sigma), pTyr1175-VEGFR2 (2478, Cell Signaling), pTyr951-VEGFR2 (2471, Cell Signaling), pTyr1054/59-VEGFR2 (44-1047G, Invitrogen), pTyr1214-VEGFR2 (AF1766, R&D Systems), VEGFR2 (9698, Cell Signaling), pTyr783-PLCγ1 (2821S, Cell Signaling), PLCγ1 (5690S, Cell Signaling), pSer1177-eNOS (9570, Cell Signaling), and eNOS (32027, Cell Signaling).

### Construction of GST-MIG6 and GST-truncated VEGFR2 domains

The cDNAs encoding human MIG6 and VEGFR2 in pCMV3 vector were obtained from SinoBiological (Beijing, China) and subcloned into pGEX-4 T-1 vector (GE Healthcare Life Sciences). The GST-fused truncated VEGFR2 domains, GST-cyto (786Leu-1356Val), GST-JX (786Leu-833Arg), GST-JX + KD1 (786Leu-929Arg), GST-KI (930Ser-1000Leu), GST-KI + KD2 (930Ser-1162Asn), and GST-C-ter (1162Asn-1356Val), were generated using a Quickchange site-directed mutagenesis kit (Agilent Technologies) according to the manufacturer’s instructions. The GST-MIG6 or truncated GST-VEGFR2 constructs were generated by PCR-based accurate synthesis with oligonucleotides (Supplementary Table 1), followed by sequencing for construct verification. The constructs were transformed into *E. coli* and the fusion proteins were purified by glutathione agarose resin.

### GST pull-down assay

For GST pull-down assay, 2 μg of GST-MIG6 fusion protein or 5 μg of each GST-VEGFR2 fusion proteins were added to 30 μl of glutathione agarose beads (sc-2009, Santa Cruz) and incubated at 4°C for 2 h. The protein bound-beads were then washed three times with NP40 buffer (P0013F, Beyotime, Shanghai, China) and incubated with the lysates of HUVECs for GST-MIG6, or lysates of HEK293T cells overexpressing Flag-tagged MIG6 for GST-VEGFR2 at 4°C for 2 h. The beads were then washed with NP40 buffer and subjected to western blot analysis.

### Antibody feeding assay

MIG6 knockdown HUVECs were confluent and starved for 4 h with serum-free media. Cells were incubated with an anti-VEGFR2 antibody (1:75 dilution, AF357, R&D Systems) at 4°C for 20 min with 0.5% FBS and rinsed with cold PBS. Cells were then incubated with serum-free media containing VEGFA (20 ng/ml) at 37°C for 30 min and rinsed twice for 2 min with cold PBS (pH 2.5). After fixation with 4% paraformaldehyde for 10 min at room temperature and permeabilization with 0.1% Triton X-100/PBS, cells were incubated with anti-Goat Alexa488 antibody (1:200 dilution, A-11055, Invitrogen) in 1% BSA/PBS for 1 h at room temperature. DAPI (D3571, Thermo Fisher Scientific) was used for nuclear staining. Internalized VEGFR2 fluorescent signals were quantified with ImageJ (Bethesda, NIH, USA) and normalized by the surface VEGFR2 fluorescent signals measured before stimulation with VEGFA.

### VEGFR2 biotinylation assay

Confluent cultures of MIG6 knockdown HUVECs were starved for 4 h with serum-free media. Cells were then incubated with EZ-Link-NHS-biotin (Thermo Fisher Scientific) in PBS for 45 min at 4°C. The reaction was quenched with 50 mM glycine in cold PBS. Then, cells were incubated with serum-free media containing VEGFA (20 ng/ml) at 37°C for indicated time points and rinsed with cold PBS twice for 15 min. Cell surface biotin was cleaved off by incubating the cells with 200 mM 2-mercaptoethane sulphonic acid (MESNA, Sigma) for 10 min on ice. After washing, cells were lysed with NP40 buffer and precipitated with streptavidin magnetic beads (Thermo Fisher Scientific) at 4°C overnight. The beads were then washed with NP40 buffer and subjected to western blot analysis for internalized VEGFR2.

### Calcium assay

MIG6 knockdown ECs were plated in EC medium with 40,000 cells/100 μl/well in a 96-well plate for overnight. An equal volume (100 μl/well) of Fluo-8 dye (ab112129, Abcam) in serum-free EC media was added. After incubation for 1 h, HHBS containing VEGFA (20 ng/ml) was added for 0, 2.5, 5, 7.5, 10, and 12.5 min. The calcium flux was monitored and recorded by the fluorescence intensity at Ex/Em = 490/525 nm.

### Electrical cell impedance sensing (ECIS)

MIG6 knockdown ECs were seeded onto gelatin-coated 8W10E ECIS array (Applied Biophysics, MA, USA) and allowed to reach confluence overnight. Following low serum starvation (1% FBS) for 3 h, permeability was measured after VEGFA treatment (50 ng/ml) using multiple frequency/time (MFT) setting. MIG6 expression was confirmed by western blot analysis.

### RhoGTPase activation assay

HUVECs treated with siControl and siMIG6 were lysed with lysis buffer (30303, NewEast Biosciences, Malvern, PA, USA). The pre-cleared cell lysates were incubated with a configuration-specific, active Rac (80501, NewEast Biosciences) or RhoA (80601, NewEast Biosciences) monoclonal antibody, and then, protein A/G agarose beads (30301, NewEast Biosciences) were added and incubated at 4°C for 1 h. Beads were washed three times, and then the bound proteins were eluted. The eluted samples were subjected to western blot analysis. Antibodies used in western blot for active RhoGTPases were: Rac1 (26005, NewEast Biosciences) and RhoA (2117T, Cell Signaling).

### In vitro permeability assay

HUVECs were grown on Matrigel (2 Matrigel:1 EC media)-coated inserts (0.4 µm polycarbonate membrane) of Transwell Permeable Supports (3413, Corning, NY, USA). After ECs became confluent, 10 µg/ml of fluorescein isothiocyanate (FITC)-dextran (70 kDa, Thermo Fisher Scientific) with VEGFA (50 ng/ml) was added into the upper chamber. Following the treatment with VEGFA for 1 h, 10 µl aliquots of media were removed from the lower chamber and diluted in 90 µl H_2_O in a 96-well plate. The fluorescence intensity with excitation at 485 nm and emission at 535 nm was measured by a microplate spectrofluorometer (Tecan, Switzerland).

### Statistical analysis

Comparisons between two groups were analyzed using paired or unpaired Student’s t-test (two-tailed), while statistical significance among four or higher number of groups was determined by one-way or two-way ANOVA with Sidak or Tukey multiple comparison test using GraphPad Prism (GraphPad Software, La Jolla, CA, USA). Data are presented as mean ± SEM from at least three independent experiments, with *p* < 0.05 being considered statistically significant.

## Results

### Vascular permeability is increased in *Mig6* knockout mice and endothelial barrier is compromised by MIG6 knockdown *in vitro*

**Fig. 1:**
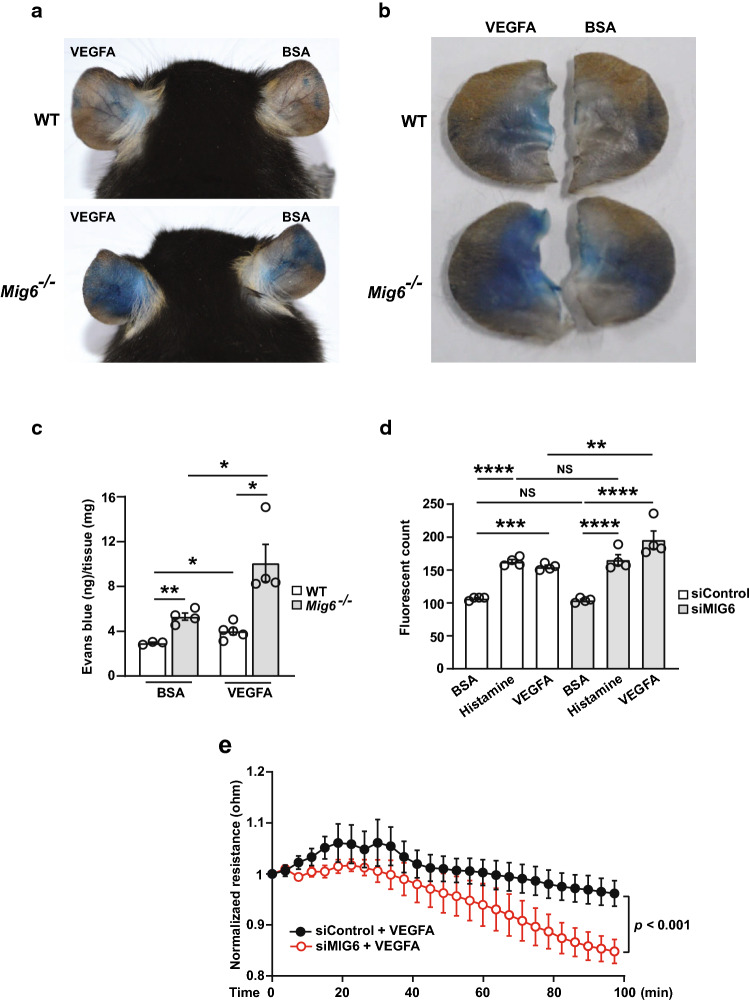
Vascular permeability is increased in *Mig6* knockout mice and endothelial barrier is compromised by MIG6 knockdown. (**a**) Representative images of WT (top) and *Mig6*^−/−^ (bottom) mouse showing Evans blue leakage, after treatment with 50 ng/ml of VEGFA (left) or BSA (right) for 1 h. (**b**) Images of Evans blue leakage in excised ears of WT and *Mig6*^−/−^ mouse. (**c**) Quantification of Evans blue leakage in ear tissues. Evans blue dye was extracted from ear skin of WT and *Mig6*^−/−^ mice to read an absorbance (*n* = 3**–**5). (**d**) Histamine (100 μM) or VEGFA (50 ng/ml)-induced EC permeability in siControl or siMIG6-treated HUVECs (*n* = 4). (**e**) Normalized resistance of siControl or siMIG6-treated ECs after stimulation with VEGFA. HUVECs were treated with siControl or siMIG6, and grown to confluence on gelatin-coated electrode arrays. After low serum starvation for 3 h, EC monolayer was stimulated with VEGFA (50 ng/ml) and TEER was measured by ECIS at a frequency of 4000 Hz. One-way ANOVA with Sidak multiple comparison test (**c**, **d)** and unpaired Student’s t-test (**e**) were performed. Data are presented as mean ± SEM. * *p* < 0.05, ** *p* < 0.01, *** *p* < 0.001, **** *p* < 0.0001, NS: not statistically significant

Although MIG6 plays critical roles in angiogenesis, it remains unknown whether it regulates vascular permeability. We therefore utilized *Mig6* knockout mice to examine this role. To investigate VEGFA-induced vascular permeability, we employed the Miles assay which measures leakage of Evans blue dye bound to albumin in mouse ears after intradermal injection of VEGFA. Increased dye leakage was observed in wild type (WT) mice after VEGFA injection compared with the BSA control group (Fig. 1a, top). Notably, BSA-injected *Mig6* knockout mice showed a higher level of basal permeability compared with WT mice, and VEGFA-injected *Mig6* knockout mice displayed a remarkable enhancement of vascular leakage (Fig. 1a, bottom). Excised mouse ears injected with VEGFA showed VEGFA-induced vessel leakage for both WT and *Mig6* knockout mice, but quantification analysis of Evans blue leakage revealed that vascular permeability was significantly increased in VEGFA-injected *Mig6* knockout mice (Fig. 1b, c). These results suggest that MIG6 may play a vital role in the maintenance of vascular integrity and alteration of MIG6 expression may lead to disruption of the blood vessel barrier. Given the enhanced level of vascular leakage observed in VEGFA-injected *Mig6* knockout mice, we next examined whether endothelial barrier is affected by MIG6 in human ECs. To address this, we evaluated EC permeability in vitro after MIG6 knockdown in ECs. The MIG6 knockdown ECs were validated first for the knockdown efficiency and responsiveness to VEGFA stimulation before the transwell permeability assay (Supplementary Fig. 1). The amount of FITC-dextran diffusing through the EC monolayer was further increased in MIG6 knockdown ECs treated with VEGFA whereas histamine-induced permeability was not significantly different between control and MIG6 knockdown cells (Fig. 1d), suggesting that MIG6 may not be involved in inflammation-induced permeability. The increased VEGFA-induced permeability in MIG6 knockdown cells was through enhanced VEGFR2 activation since pretreatment with Axitinib, a tyrosine kinase inhibitor that selectively targets VEGFRs (Liu et al. 2022), attenuated the increase in permeability in MIG6 knockdown cells (Supplementary Fig. 2a). EC barrier function was also assessed by transendothelial electrical resistance (TEER) using an electric cell-substrate impedance sensor (ECIS), which can monitor the barrier integrity in real-time. There was a delay in reaching the same stabilized TEER in MIG6 knockdown cells compared with control counterparts before treatment with VEGFA (Supplementary Fig. 2b). Upon VEGFA stimulation, the disrupted endothelial barrier by weakened cell–cell junctions allowed the current (4000 Hz) to pass more freely through the layer, consequently leading to a more prominent decrease in electrical resistance in MIG6 knockdown cells (Fig. 1e). Together, these data demonstrated that MIG6 knockdown promotes EC permeability.

### MIG6 expression is upregulated by VEGFA and suppressed by VEGFR2 inhibition in endothelial cells

**Fig. 2:**
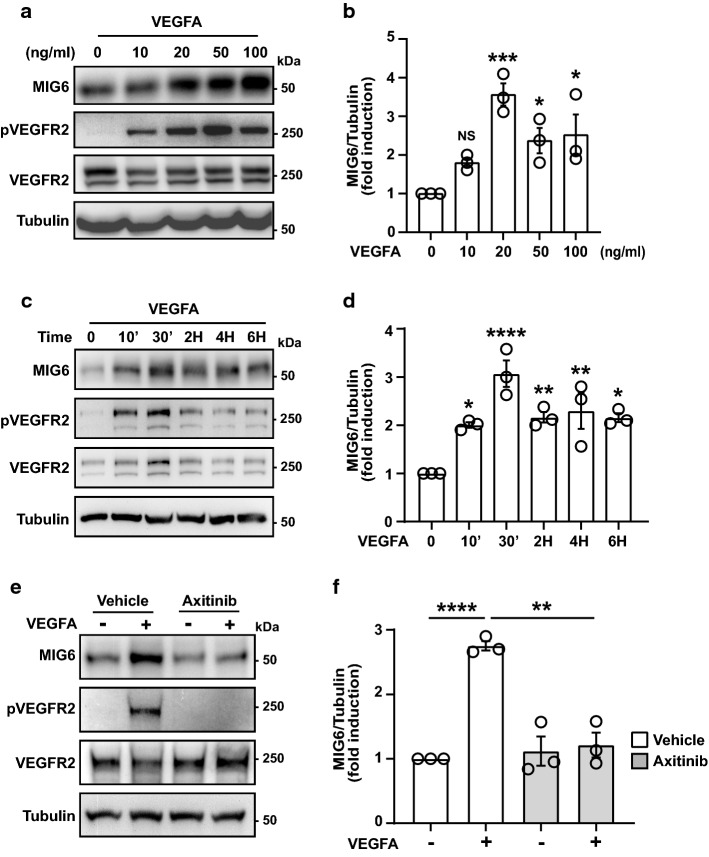
MIG6 expression is induced by VEGFA and suppressed by VEGFR2 inhibition in ECs. (**a**) VEGFA-induced MIG6 expression. Serum-starved HUVECs were stimulated with a different amount of VEGFA for 30 min. VEGFR2 activation was monitored by phosphorylation of Tyr1175. (**b**) MIG6 induction was analyzed by densitometry and normalized by tubulin. (**c**) Time kinetic assay of MIG6 induction in HUVECs. Serum-starved ECs were stimulated with VEGFA (20 ng/ml) for different time points. (**d**) MIG6 induction at different time points was measured by densitometry and normalized by tubulin. (**e**) HUVECs were treated with a VEGFR2 tyrosine kinase inhibitor Axitinib (10 nM) for 3 h prior to VEGFA treatment (20 ng/ml) for 30 min. The diminished VEGFA-induced MIG6 expression is shown by western blot. (**f**) MIG6 induction was quantified by densitometry and normalized by tubulin. Fold induction relative to the control is shown as the mean ± SEM (*n* = 3 for **b**, **d**, and **f**). Statistical significance was determined by one-way ANOVA with Sidak multiple comparison test. * *p* < 0.05, ** *p* < 0.01, *** *p* < 0.001, **** *p* < 0.0001, NS: not statistically significant

A number of growth factors including epidermal growth factor (EGF) and hepatocyte growth factor (HGF) have been reported to induce MIG6 (Pante et al. 2005; Zhang and Vande Woude 2007). However, it remains thus far unknown whether MIG6 is induced by VEGFA. In addition, given that vascular permeability is disrupted by *Mig6* deficiency, we further investigated whether VEGFA can regulate MIG6 expression. We found that MIG6 protein expression was increased in ECs upon VEGFA stimulation (Fig. 2a, b). In addition, VEGFA-induced MIG6 expression was confirmed by the time kinetic assay (Fig. 2c, d). Notably, MIG6 protein expression was promptly upregulated as early as 10 min after treatment with VEGFA and sustained up to 6 h. We then evaluated whether VEGFA-induced MIG6 expression requires VEGFR2 activation by treating cells with a VEGFR inhibitor Axitinib. VEGFA treatment induced MIG6 expression and increased VEGFR2 phosphorylation in ECs, but Axitinib treatment attenuated the VEGFA-induced MIG6 induction and VEGFR2 phosphorylation, suggesting that VEGFR2 activation is required for MIG6 induction by VEGFA (Fig. 2e, f). Collectively, these data showed that MIG6 is induced as an early responder to VEGFA and its induction is decreased by VEGFR2 inhibition in ECs.

### MIG6 binds to VEGFR2 in endothelial cells

**Fig. 3:**
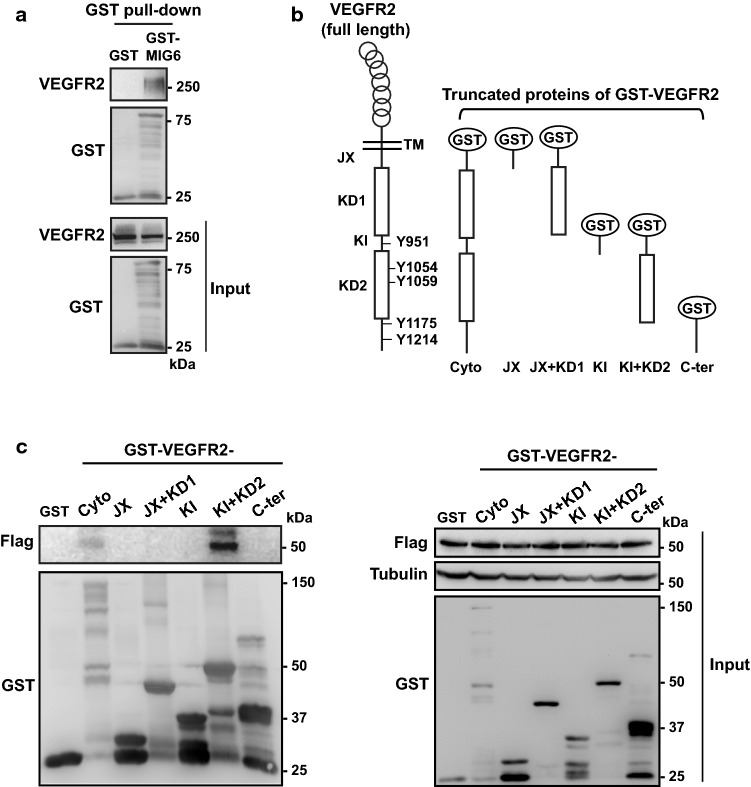
MIG6 binds to VEGFR2. (**a**) Association of MIG6 with VEGFR2 was assessed by GST pull-down assay, followed by western blot. (**b**) Schematic representation of VEGFR2 protein domains and the truncated proteins of GST-fusion VEGFR2 domains. Y951, Y1054, Y1059, Y1175, and Y1214 are major tyrosine phosphorylation sites located in the indicated domains of VEGFR2. TM, transmembrane; JX, juxtamembrane domain; KD1, kinase domain 1; KI, kinase insert domain; KD2, kinase domain 2; Cyto, whole cytoplasmic domain of VEGFR2; C-ter, C-terminus domain of VEGFR2 excluding JX, KD1, KI, and KD2. (**c**) Association of Flag-tagged MIG6 with truncated VEGFR2 proteins was assessed by GST pull-down assay, displaying that MIG6 binds to the cytoplasmic domain and kinase domain 2 of VEGFR2. Inputs are shown on the right

It was reported that MIG6 binds to all four members of EGFR/ErbB and inhibits EGFR autophosphorylation in vitro (Anastasi et al. 2016; Xu and Li 2021). Moreover, MIG6 binds to the EGFR kinase domain, which results in suppression of EGFR kinase activity (Ferby et al. 2006; Hackel Peter et al. 2001). As MIG6 is induced by VEGFA in ECs, we tested whether MIG6 binds to VEGFR2 and affects its activation. To examine MIG6 binding to VEGFR2, GST-MIG6 fusion protein was utilized (Liu et al. 2021). The GST pull-down assay revealed that MIG6 binds to the full-length VEGFR2 (Fig. 3a). Furthermore, we generated GST-fused VEGFR2 mutant proteins to determine the MIG6 binding domain of VEGFR2 (Fig. 3b). The GST pull-down assay showed that Flag-tagged MIG6 protein binds to the entire cytoplasmic domain (Cyto) and tyrosine kinase domain 2 (KD2) of VEGFR2, but not to juxtamembrane domain (JX), tyrosine kinase domain 1 (KD1), and kinase insert domain (KI) (Fig. 3c), suggesting that MIG6 may regulate critical activities of VEGFR2 by binding to the VEGFR2 KD2.

### MIG6 knockdown increases VEGFR2 phosphorylation and internalization in response to VEGFA

**Fig. 4:**
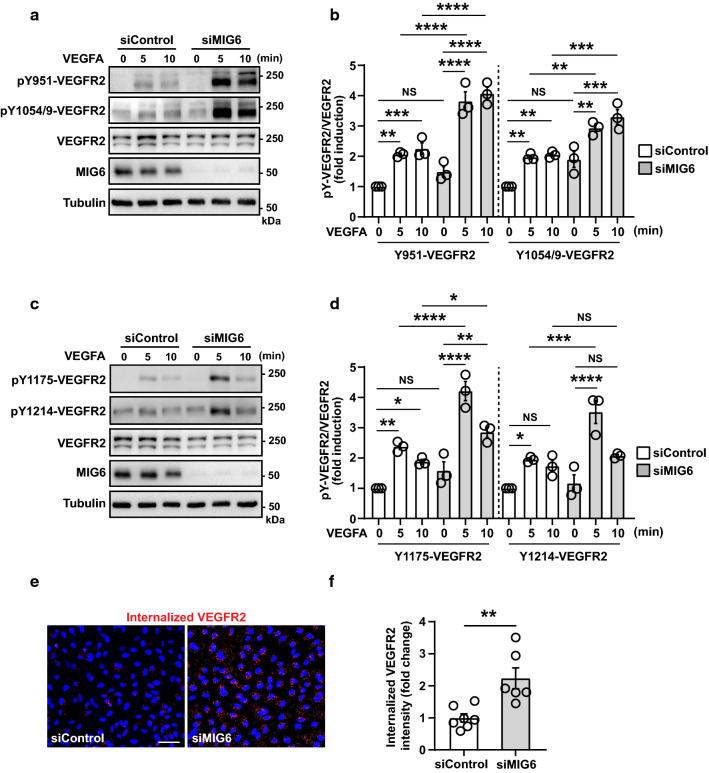
Tyrosine phosphorylation and internalization of VEGFR2 are upregulated in MIG6 knockdown ECs. (**a, c**) MIG6 knockdown in HUVECs increases VEGFR2 phosphorylation on Tyr951 and Tyr1054/9 (**a)**, and Tyr1175 and Tyr1214 (**c**) in response to VEGFA (50 ng/ml) at 5 and 10 min. (**b**, **d**) Tyrosine phosphorylation of VEGFR2 shown in (**a**) and (**c**) was analyzed and normalized by total VEGFR2. Fold induction relative to the control is shown as the mean ± SEM (*n* = 3 for **b** and **d**). (**e**) Antibody feeding assay of VEGFR2 internalization (red fluorescence) in response to VEGFA (20 ng/ml) for 30 min in ECs treated with siControl and siMIG6 (*n* = 6–7). (**f**) Quantification of internalized VEGFR2 fluorescence in (**e**). The internalized fluorescence intensity was analyzed and normalized by the surface level of VEGFR2. Data are presented as mean ± SEM. One-way ANOVA with Sidak multiple comparison test (**b**, **d**) and unpaired Student’s t-test (**f**) were used for statistical analyses. * *p* < 0.05, ** *p* < 0.01, *** *p* < 0.001, **** *p* < 0.0001, NS: not statistically significant

Given the MIG6 binding to VEGFR2, we explored VEGFR2 phosphorylation changes in MIG6 knockdown ECs by employing phospho-specific VEGFR2 antibodies. We found that MIG6 knockdown strongly enhanced VEGFR2 phosphorylation on Y951 and Y1054/9 upon VEGFA stimulation (Fig. 4a, b). Similarly, phosphorylation on Y1175 and Y1214 was increased by VEGFA in MIG6 knockdown ECs (Fig. 4c, d). Furthermore, MIG6 overexpression in ECs decreased VEGFR2 phosphorylation in response to VEGFA (Supplementary Fig. 3a-f). These data demonstrate that MIG6 has an inhibitory effect on VEGFR2 phosphorylation and its kinase activity by binding to the KD2 of VEGFR2. Since MIG6 negatively regulates VEGFR2 phosphorylation, we assessed whether MIG6 also affects VEGFR2 internalization which can regulate vascular permeability due to its close association with maximal VEGFR2 activation and signaling (LeBlanc et al. 2019; Tian et al. 2016). To address this, we used an antibody feeding assay in which the extracellular domain of VEGFR2 is labeled with an antibody to enable the visualization of VEGFR2 endocytosis and trafficking. MIG6 knockdown ECs showed an increase in VEGFA-induced VEGFR2 internalization (Fig. 4e, f). This finding was also supported by a biotinylation assay for VEGFR2 internalized from the cell surface (Supplementary Fig. 4), demonstrating that VEGFA-induced VEGFR2 internalization in ECs is enhanced by MIG6 knockdown.

### MIG6 knockdown activates the signaling axis of PLCγ1-Ca^***2***+^-eNOS and disrupts the balance of RAC1/RHOA GTPase activation

**Fig. 5:**
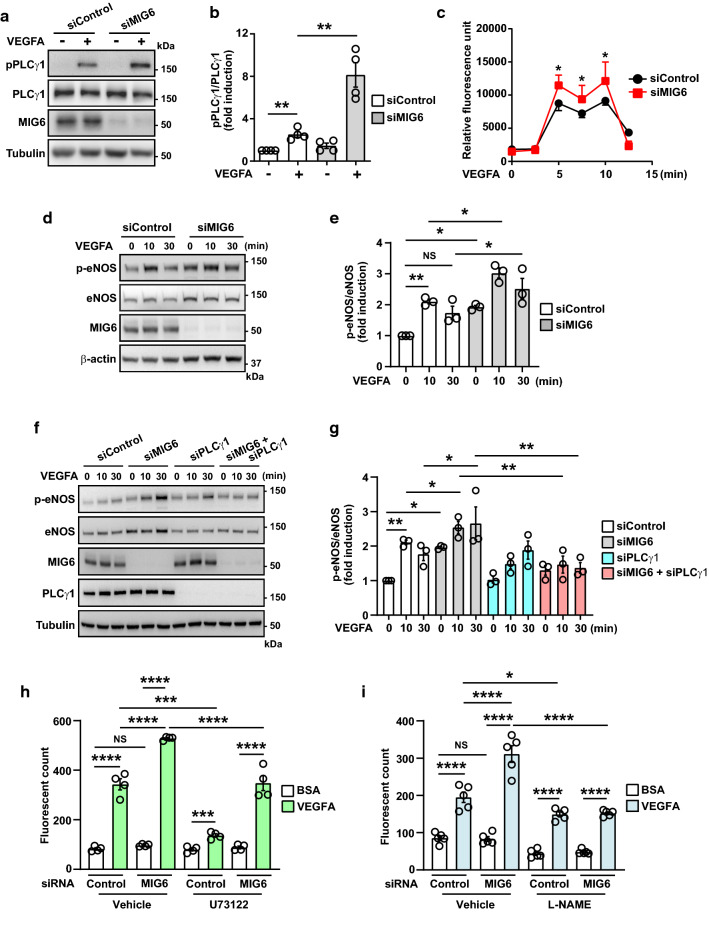
MIG6 knockdown activates PLCγ1-Ca^2+^-eNOS signaling and VEGFA-induced increase of permeability by MIG6 knockdown is attenuated by PLCγ1 and eNOS inhibitors. (**a**) Western blot analysis of PLCγ1 phosphorylation on Tyr783 in siControl and siMIG6-treated ECs in response to VEGFA (50 ng/ml) at 10 min. (**b**) PLCγ1 phosphorylation on Tyr783 was analyzed and normalized by total PLCγ1 (*n* = 4). (**c**) Intracellular Ca^2+^ level in serum-starved ECs treated with siControl and siMIG6 was determined in response to VEGFA (20 ng/ml) at various time points (*n* = 3). (**d**) Western blot analysis of eNOS phosphorylation on Ser1177 in ECs treated with siControl and siMIG6, after treatment with VEGFA (50 ng/ml) for 10 min and 30 min. (**e**) eNOS phosphorylation on Ser1177 was analyzed and normalized by total eNOS (*n* = 3). (**f**) Western blot analysis of eNOS phosphorylation on Ser1177 in ECs treated with siControl, siMIG6, siPLCγ1, and siMIG6 + siPLCγ1, followed by treatment with VEGFA (50 ng/ml) for 10 min and 30 min. (**g**) eNOS phosphorylation on Ser1177 was analyzed and normalized by total eNOS (*n* = 3). (**h, i**) VEGFA-induced permeability was measured in siControl or siMIG6 knockdown ECs treated with a PLCγ1 inhibitor (U73122, 3 μM) for 30 min or an eNOS inhibitor (L-NAME, 300 μM) for 1 h prior to VEGFA (50 ng/ml) treatment (*n* = 4 for **h**; *n* = 5 for **i**). Statistical significance was determined by one-way ANOVA with Sidak multiple comparison test (**b**, **e**, and **g)**, paired Student’s t-test (**c**), and two-way ANOVA with Tukey multiple comparison test (**h, i)**. Data are presented as mean ± SEM. * *p* < 0.05, ** *p* < 0.01, *** *p* < 0.001, **** *p* < 0.0001, NS: not statistically significant

Various signaling molecules including PLCγ1, eNOS, and RhoGTPases are implicated in the regulation of vascular permeability (Chen and Simons 2021; Flentje et al. 2019; Shu et al. 2015). Given that VEGFR2 phosphorylation on Y1175 is increased by MIG6 knockdown, we examined PLCγ1 and eNOS phosphorylation to determine whether MIG6 affected the signaling axis of PLCγ1 and eNOS to cause an increase in VEGFA-induced vascular permeability in MIG6 knockdown cells. PLCγ1 phosphorylation in response to VEGFA was further increased in MIG6 knockdown cells (Fig. 5a, b), and consequently, cytosolic Ca^2+^ level was transiently elevated (Fig. 5c). We also found that MIG6 knockdown markedly increased the phosphorylation of eNOS (Fig. 5d, e), which was reversed by co-depletion of PLCγ1 (Fig. 5f, g). Consistently, pretreatment with a PLCγ1 inhibitor U73122 or an eNOS inhibitor L-NAME significantly attenuated the increase of VEGFA-induced permeability in MIG6 knockdown cells (Fig. 5h, i). Taken together, these data establish the role of the PLCγ1-Ca^2+^-eNOS axis in MIG6-mediated regulation of vascular permeability in response to VEGFA.

**Fig. 6:**
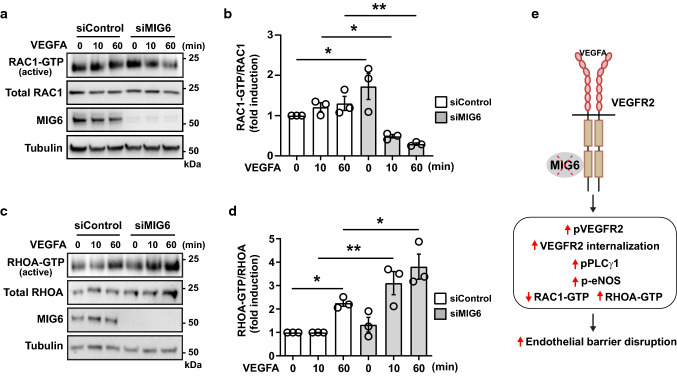
The balance of RAC1/RHOA GTPase activation is disrupted in MIG6 knockdown ECs. (**a, c**) Western blot analysis of RAC1-GTPase (**a)** and RHOA-GTPase (**c)** activity in siControl and siMIG6 knockdown ECs treated with VEGFA (50 ng/ml) for 10 and 60 min. (**b**, **d**) RAC1-GTPase (**b)** and RHOA-GTPase (**d)** activation was analyzed and normalized by total RAC1 and RHOA, respectively. (**e**) The proposed working model illustrates that MIG6 deficiency promotes VEGFA-induced vascular permeability via activation of PLCγ1 and eNOS signaling and perturbation of the balance in RAC1/RHOA activation, resulting in endothelial barrier disruption. One-way ANOVA with Sidak multiple comparison test was performed (**b**, **d)**. Data are presented as mean ± SEM (*n* = 3 for **b, d**). * *p* < 0.05, ** *p* < 0.01

The integrity of EC barrier is maintained by the fine balance between RHOA and RAC1 activation (Pronk et al. 2019; Radeva and Waschke 2018). When RHOA activation is increased but RAC1 activity is antagonized, this alteration leads to junction destabilization and consequent increase in endothelial permeability. We therefore asked whether MIG6 knockdown compromised the endothelial barrier by modulation of RAC1 and RHOA activation. VEGFA-induced RAC1 activation was found to be decreased but RHOA activation was markedly increased in MIG6 knockdown ECs (Fig. 6a–d). Furthermore, augmentation of RAC1 activity by lentiviral overexpression of constitutively active RAC1 (Q61L) mutant (Burstein et al. 1998) in MIG6 knockdown cells significantly reduced VEGFA-induced permeability to the level of control knockdown cells (Supplementary Fig. 5). These results demonstrate that MIG6 knockdown may disrupt the endothelial barrier function by perturbing the balance between RAC1 and RHOA activation.

## Discussion

Abnormal blood vessel growth with vascular hyperpermeability promotes the development of neovascular diseases. An increase in vascular permeability precedes the onset of neovascularization and is a hallmark of pathological angiogenesis (Bates 2010). Previously, we found that *Mig6* knockout mice displayed increased blood vessel density and number of branch points in the retinae, demonstrating an anti-angiogenic effect of Mig6 in neovascularization (Liu et al. 2021). However, it remains unknown whether MIG6 regulates vascular permeability. In this study, we showed that vascular permeability was increased in *Mig6* knockout mice and MIG6 knockdown elevated VEGFA-induced endothelial barrier breakdown in vitro. At the molecular level, we found that MIG6 was induced by VEGFA and bound to the VEGFR2 kinase domain 2, which blocked VEGFA-induced VEGFR2 phosphorylation. We also revealed that the signaling axis of PLCγ1-Ca^2+^-eNOS was further activated and contributed to the endothelial barrier disruption upon MIG6 knockdown in ECs.

Vascular permeability is greatly increased in diverse pathophysiological conditions such as inflammation, wound healing, and cancers (Claesson-Welsh et al. 2021; Dvorak 2019; Hellenthal et al. 2022). Accordingly, endothelial barrier functions are regulated by various permeability inducers including histamine, thrombin, and VEGFA, depending on the pathophysiological contexts (Park-Windhol and D'Amore 2016; Wautier and Wautier 2022; Wettschureck et al. 2019). The early onset osteoarthritis condition observed in *Mig6* knockout mice (Zhang et al. 2005) suggested the possibility that inflammatory conditions may be driven by *Mig6* deficiency and be involved in the development of the joint disease and the minor increase in vascular leakage in the absence of external stimuli; however, no significant infiltration of inflammatory cells was observed in the diseased joints of the mice and further ablation of *Rag2* gene did not rescue the joint phenotype of the *Mig6*-deficient mice (Zhang et al. 2005). This suggests that inflammatory conditions and the immune system may not be critically involved in the increased basal permeability observed in *Mig6* knockout mice. Consistent with the notion, we found that histamine-induced permeability was not affected by MIG6 knockdown in ECs. Another relevant phenotype displayed by the *Mig6*-deficient mice with regard to vascular permeability is that these mice show pro-angiogenic characteristics demonstrated by the increased blood vessel density and branch points in the retinae (Liu et al. 2021). As regulation of vascular permeability is closely associated with angiogenic processes implicating VEGFR2 activation and the signaling pathways leading to different VEGFR2-mediated biological effects often overlap or crosstalk with each other (Claesson-Welsh et al. 2021; Simons et al. 2016), it is highly likely that anti-angiogenic functions exerted by MIG6 can impact on VEGFA-induced vascular permeability as well. The delay in reaching the same TEER in MIG6 knockdown cells compared with control counterparts may reflect the effect of altered angiogenesis on vascular permeability although the underlying mechanisms remain to be elucidated. We previously demonstrated that anti-angiogenic functions of MIG6 are mediated by binding to SHC1 and restraining its phosphorylation (Liu et al. 2021). Given that SHC1 is a common adaptor protein transducing diverse signals derived from activation of many receptor tyrosine kinases including VEGFR2 (Oshikawa et al. 2012; Wills and Jones 2012) we attempted to examine whether SHC1 depletion affects VEGFA-induced vascular permeability and how this would alter the increased permeability observed in MIG6 knockdown cells. Both single SHC1 knockdown and double SHC1/MIG6 knockdown ECs showed a comparable increase in permeability to the level of single MIG6 knockdown cells upon VEGFA stimulation (data not shown). At present, we are unable to interpret this observation clearly, but we speculate that the permeability changes in SHC1 knockdown cells may reflect an effect of more complicated and fundamental alterations in signaling due to the depletion of SHC1, a common signal transducer for tyrosine kinase receptors. Nonetheless, this suggests that VEGFA-induced regulation of vascular permeability may be significantly affected by the complex interplay among signaling molecules and pathways involved in other VEGFR2-mediated biological effects.

We showed for the first time that MIG6 regulates VEGFR2 phosphorylation by binding to VEGFR2 kinase domain 2. Depletion of MIG6 in ECs significantly increased VEGFA-induced phosphorylation at multiple sites of VEGFR2, indicating that various biological events mediated by VEGFR2 phosphorylation might be affected by MIG6 knockdown. The effects of MIG6 on phosphorylation of multiple tyrosine residues were also observed in the case of EGFR in which the first segment (337Ser-361Ser) of EGFR binding domain (EBD) in MIG6 was proposed to bind the EGFR kinase domain (Xu and Li 2021; Zhang et al. 2007) and MIG6 overexpression attenuated the phosphorylation of Tyr1068 and Tyr1173 on EGFR (Bellini et al. 2020; Descot et al. 2009; Liu et al. 2012). Given the regulatory role of MIG6 in the overall phosphorylation of these receptor kinases, it is possible that any biological effect observed in MIG6 knockdown cells may derive from a combination of signaling pathways involving multiple phosphorylation sites of the receptor. Nevertheless, some phosphorylation sites appear to have more defined functions and signaling pathways leading to the biological effect (Koch et al. 2011; Wang et al. 2020). For example, Tyr1175 (Y1175) is a major phosphorylation site on VEGFR2 to activate PLCγ1 (Takahashi et al. 2001). The signaling cascade of VEGFR2-PLCγ1-Ca^2+^-eNOS is a crucial pathway to control endothelial barrier function (Dalal et al. 2020; Dragoni et al. 2021; Wu et al. 1999). In addition, phosphorylated Y1175 (pY1175) of VEGFR2 binds to SHC1, SHB, and SCK to activate further downstream signaling (Holmqvist et al. 2004; Warner et al. 2000), and has been proposed to play a critical role in VEGFA-induced vascular hyperpermeability (Hendel et al. 2014; Kim et al. 2019; Yang et al. 2017). In this study, we demonstrated that VEGFA further increased VEGFR2 phosphorylation on Y1175 that augmented PLCγ1 activation in MIG6 knockdown cells. Furthermore, PLCγ1 inhibition attenuated VEGFA-induced permeability in MIG6 knockdown cells and PLCγ1 depletion in MIG6 knockdown cells significantly decreased eNOS phosphorylation in response to VEGFA. These results altogether support the role of pY1175 in mediating the effects of MIG6 knockdown on the increased permeability at least in part. Furthermore, as a small signaling mediator, Ca^2+^ is an important signaling effector which affects vascular permeability (Wu et al. 1999). VEGFA-induced acute vascular leakage was associated with upregulation of PLCγ1 phosphorylation and eNOS phosphorylation by transiently increased intracellular calcium concentration ([Ca^2+^]_i_) (Dalal et al. 2020)_._ VEGFA-induced vascular permeability implicates increased eNOS activity, and phosphorylation of eNOS at several sites is critical for the eNOS activity to regulate endothelial barrier function (Shu et al. 2015). Consistent with our results regarding the involvement of pY1175 VEGFR2 in the regulation of vascular permeability upon MIG6 depletion, the transient increase of [Ca^2+^]_i_ was markedly augmented by VEGFA-induced PLCγ1 phosphorylation, which in turn led to the robust phosphorylation of eNOS. In this study, we have presented the pY1175 VEGFR2-PLCγ1-eNOS axis as one of the major signaling pathways affected by MIG6 status regarding vascular permeability among diverse VEGFR2 activation-mediated biological events. Given the multiple molecules involved in the regulation of vascular permeability (Claesson-Welsh et al. 2021; Komarova et al. 2017), it would be interesting to investigate the function of MIG6 in other VEGFR2-mediated signaling pathways to clearly define the downstream pathways contributing to MIG6 deficiency-driven alteration of endothelial barrier function.

In summary, we demonstrate that MIG6 deficiency increases VEGFA-induced vascular permeability in vivo and disrupts endothelial barrier integrity in vitro. We also unraveled that MIG6 regulates vascular permeability by binding to VEGFR2 kinase domain 2, which involves the signaling cascade of PLCγ1-Ca^2+^-eNOS and perturbation of RAC1/RHOA activation upon MIG6 deficiency to result in disrupted endothelial barrier (Fig. 6e). Therefore, our findings support the critical function of MIG6 in the homeostasis of vascular permeability by antagonizing VEGFR2 activation and downstream signaling pathways, with an implication of MIG6 as an applicable target for modulation of VEGFA-induced permeability under pathological conditions.

## Supplementary Information

Below is the link to the electronic supplementary material.

Supplementary file1 (PDF 393 kb)
