# Correction to: Critical role of mitogen-inducible gene 6 in restraining endothelial cell permeability to maintain vascular homeostasis

**DOI:** 10.1007/s12079-022-00709-8

**Published:** 2022-12-07

**Authors:** Liying Xing, Guanqun Huang, Rongyuan Chen, Lijuan Huang, Juanxi Liu, Xiangrong Ren, Shasha Wang, Haiqing Kuang, Anil Kumar, Jong Kyong Kim, Qin Jiang, Xuri Li, Chunsik Lee

**Affiliations:** 1 State Key Laboratory of Ophthalmology, Zhongshan Ophthalmic Center, Guangdong Provincial Key Laboratory of Ophthalmology and Visual Science, Sun Yat-sen University, 510060 Guangzhou, China; 2 Affiliated Eye Hospital of Nanjing Medical University, 210000 Nanjing, China

**Correction to: J Cell Commun Signal**

10.1007/s12079-022-00704-z

The article “Critical role of mitogen-inducible gene 6 in restraining endothelial cell permeability to maintain vascular homeostasis”, written by Liying Xing, Guanqun Huang, Rongyuan Chen, Lijuan Huang, Juanxi Liu, Xiangrong Ren, Shasha Wang, Haiqing Kuang, Anil Kumar, Jong Kyong Kim, Qin Jiang, Xuri Li and Chunsik Lee, was originally published electronically on the publisher’s internet portal on 25 October 2022 without open access. With the author(s) decision to opt for Open Choice the copyright of the article changed on 31 October 2022 to © The Author(s) 2022 and the article is forthwith distributed under a Creative Commons Attribution.

This article is licensed under a Creative Commons Attribution 4.0 International License, which permits use, sharing, adaptation, distribution and reproduction in any medium or format, as long as you give appropriate credit to the original author(s) and the source, provide a link to the Creative Commons licence, and indicate if changes were made.

The images or other third party material in this article are included in the article’s Creative Commons licence, unless indicated otherwise in a credit line to the material. If material is not included in the article’s Creative Commons licence and your intended use is not permitted by statutory regulation or exceeds the permitted use, you will need to obtain permission directly from the copyright holder.

To view a copy of this licence, visit http://creativecommons.org/licenses/by/4.0.

