# Supplementary material for: Critical role of mitogen-inducible gene 6 in restraining endothelial cell permeability to maintain vascular homeostasis
Source: J Cell Commun Signal. 2022 Oct 25;17(1):151–65. doi: 10.1007/s12079-022-00704-z (PMC10030747; doi:10.1007/s12079-022-00704-z)
Supplement: Supplementary file 1 — Supplementary file1 (PDF 393 kb) [file 12079_2022_704_MOESM1_ESM.pdf]

Critical role of Mitogen-inducible gene 6 in restraining endothelial cell permeability to maintain vascular homeostasis

Liying Xing[1], Guanqun Huang[1], Rongyuan Chen[1], Lijuan Huang[1], Juanxi Liu[1], Xiangrong Ren[1], Shasha Wang[1], Haiqing Kuang[1], Anil Kumar[1], Jong Kyong Kim[1], Qin Jiang[2]*, Xuri Li[1]*, Chunsik Lee[1]*

[1]State Key Laboratory of Ophthalmology, Zhongshan Ophthalmic Center, Sun Yat-sen University, Guangdong Provincial Key Laboratory of Ophthalmology and Visual Science, Guangzhou 510060, China

[2]Affiliated Eye Hospital of Nanjing Medical University, Nanjing 210000, China

* Correspondence:
Chunsik Lee: chunsik@mail.sysu.edu.cn
Xuri Li: lixr6@mail.sysu.edu.cn
Qin Jiang: jqin710@vip.sina.com

Supplementary Information

Supplementary Table 1 siRNA and primer sequences used in this study

**siRNA sequences**	
siMIG6 #1	5’-CUACACUUUCUGAUUUCAA-3’
siMIG6 #2	5’-GCAGGGUAUCCAUUCUUUA-3’
siMIG6 #3	5’-GGAUAUCCAACUGUUGUAU-3′
siPLCγ1	5'-GUACUGUGUUUCGCAUUAA-3'
**Primers for GST-fused truncation mutants**	
GST-VEGFR2 cyto	Forward: CGGAATTCCTGCGTACCGTG<br>Reverse: CGCTCGAGTTAAACCGGCGG
GST-VEGFR2 JX	Forward: CGGAATTCCTGCGTACCGTGAAAC<br>Reverse: CGCTCGAGTTAGCGATCGCG
GST-VEGFR2 JX + KD1	Forward: CGGAATTCTTACGCACCGTGA<br>Reverse: CACTCGAGTTAACGCAGATAGG
GST-VEGFR2 KI	Forward: CCGGAATTCAGCAAACGCAATG<br>Reverse: CCGCTCGAGTTACAGGAAGTC
GST-VEGFR2 KI + KD2	Forward: CCGAATTCTCTAAACGTAATG<br>Reverse: GCCTCGAGTTAATTACCCAG
GST-VEGFR2 C-ter	Forward: CGGAATTCCTGCTGCAGGCA<br>Reverse: CCGCTCGAGTTACACCGGCGG

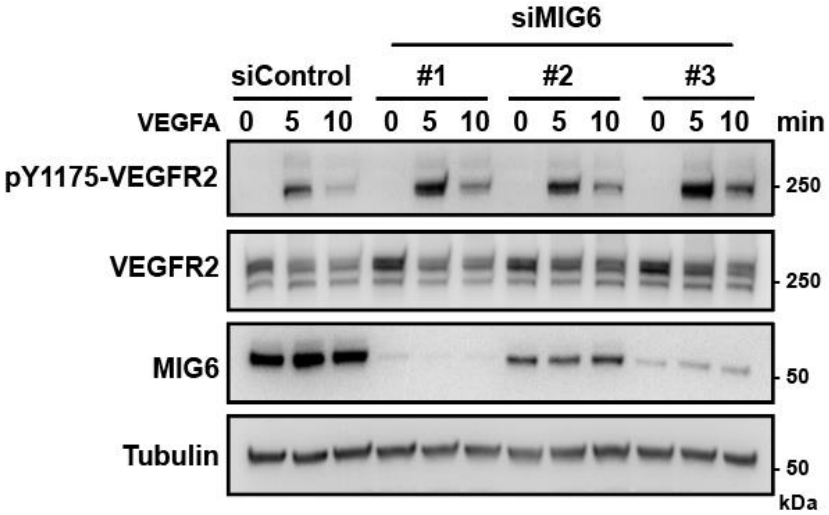

Supplementary Fig. 1 MIG6 expression changes after MIG6 knockdown in ECs. Western blot analysis for MIG6 knockdown efficiency and responsiveness to VEGFA stimulation (50 ng/ml) is shown for ECs transfected with three different MIG6 siRNAs

a

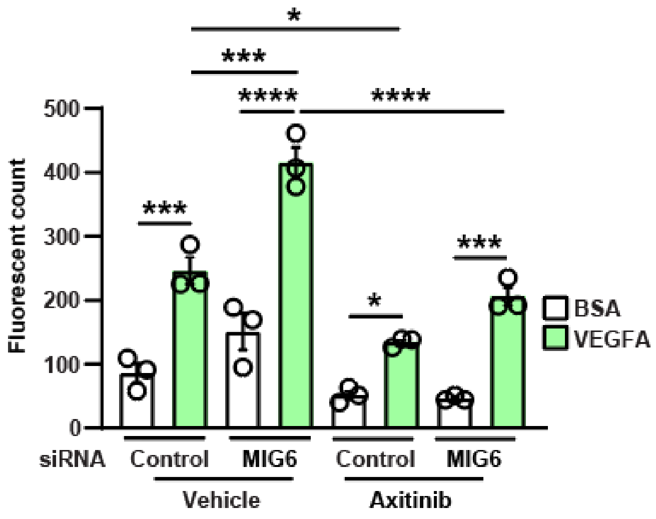

b

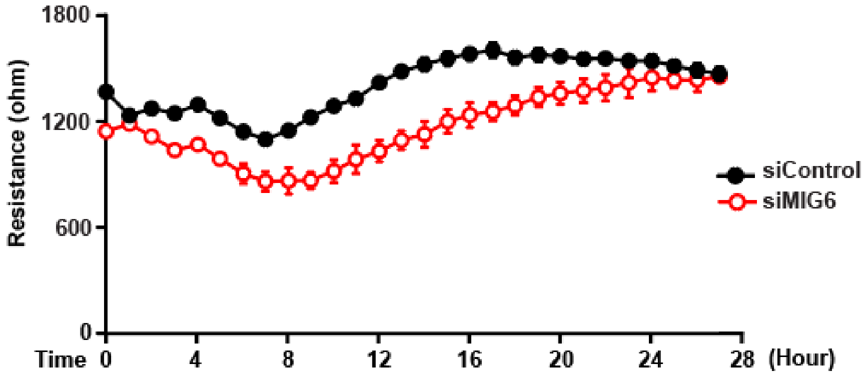

Supplementary Fig. 2 Effects of MIG6 knockdown on endothelial permeability and barrier function. (**a**) Enhanced permeability in MIG6 knockdown ECs is attenuated by VEGFR2 inhibition. VEGFA-induced permeability was measured in siControl or siMIG6 knockdown ECs treated with a VEGFR2 inhibitor (Axitinib, 1 μM) for 1 hr prior to VEGFA (50 ng/ml) treatment (n = 3). (**b**) HUVECs were treated with siControl or siMIG6, and grown to confluence on gelatin-coated electrode arrays. TEER before VEGFA stimulation measured at a frequency of 4000 Hz by ECIS over the indicated time is shown without normalization. Two-way ANOVA with Tukey multiple comparison test was performed (**a**). Data are presented as mean ± SEM. * $p < 0.05$, *** $p < 0.001$, **** $p < 0.0001$

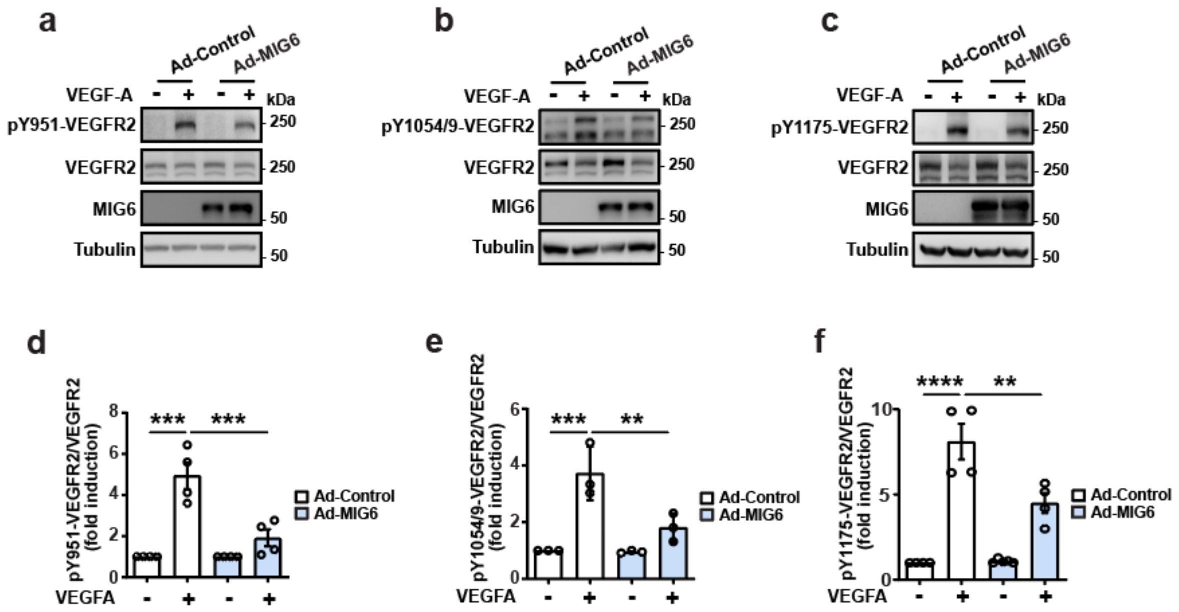

Supplementary Fig. 3 MIG6 overexpression inhibits VEGFR2 phosphorylation in ECs. (**a-c**) Adenovirally overexpressed MIG6 in HUVECs decreased VEGFR2 phosphorylation on Tyr951 (**a**), Tyr1054/9 (**b**), and Tyr1175 (**c**) in response to VEGFA stimulation (50 ng/ml) for 30 min. (**d-f**) Phosphorylation of VEGFR2 on Tyr951 (**d**), Tyr1054/9 (**e**), and Tyr1175 (**f**) was analyzed and normalized by total VEGFR2 (n = 3-4 for **d-f**). Fold induction relative to the control is shown as the mean ± SEM. One-way ANOVA with Sidak multiple comparison test was performed (**d-f**). ** $p < 0.01$, *** $p < 0.001$, **** $p < 0.0001$

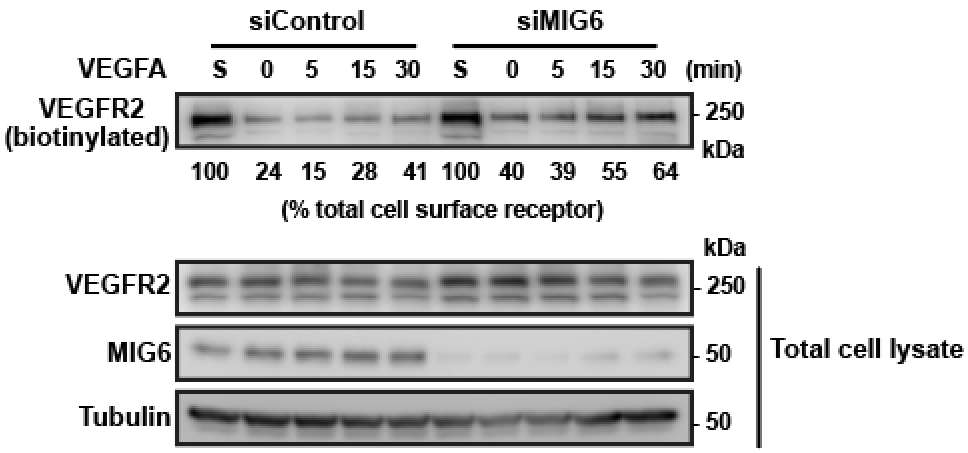

Supplementary Fig. 4 MIG6 knockdown increases VEGFR2 internalization. Biotinylation assay for VEGFR2 internalization from the cell surface was performed at different time points in response to VEGFA (20 ng/ml). The first lane of each cell group (S) indicates the biotinylated VEGFR2 level on the cell surface without VEGFA stimulation prior to cleaving off cell surface biotin, and this original VEGFR2 level on the cell surface was used to determine the relative abundance of internalized VEGFR2 in the streptavidin precipitates after VEGFA treatment as indicated

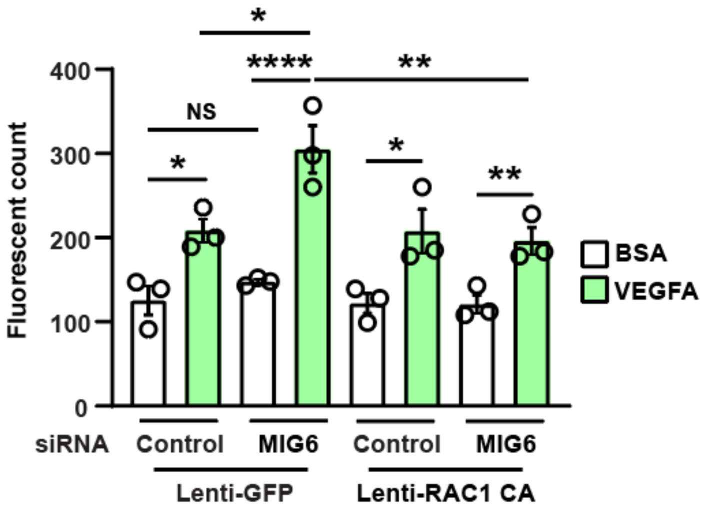

Supplementary Fig. 5 VEGFA-induced permeability in MIG6 knockdown cells is decreased by the overexpression of constitutively active (CA) RAC1 mutant. Control and MIG6 knockdown cells were transduced with lenti-GFP or lenti-RAC1 CA (Q61L) mutant viruses. VEGFA-induced permeability was measured in the knockdown cells overexpressing GFP or RAC1 CA (n = 3). Statistical significance was determined by two-way ANOVA with Tukey multiple comparison test. Data are presented as mean ± SEM. * $p < 0.05$, ** $p < 0.01$, **** $p < 0.0001$, NS: not statistically significant
